# Supplementary material for: Divergent patterns of richness and density in the global soil seed bank
Source: Nat Commun. 2026 Aug 22;17:8820. doi: 10.1038/s41467-026-77079-x (PMC13499811; doi:10.1038/s41467-026-77079-x)
Supplement: Supplementary file 1 [file 41467_2026_77079_MOESM1_ESM.pdf]

Supplementary Information

Divergent patterns of richness and density in the global soil seed bank

Contents

Table S1. Summary of the Global Seed Bank Database
Table S2. Details on each univariate hierarchical mixed effects model for main analysis
Table S3. Richness-area slopes
Table S4. Average soil seed bank densities per realm
Table S5. Summary of model estimates of seed density (m^{-2})
Table S6. Predicted estimates of diversity at different scales

Figure S1. Model fit for species richness models
Figure S2. Model fit for seed density models
Figure S3. Species richness in the soil seed bank as a function of total soil samples (m^2)

Table S1. Summary of the Global Seed Bank Database. Number of studies checked for data, total number of studies with usable data, total number of records, and observations. By 'observation', we mean a single value for a metric describing the seed bank community (such as richness, density or abundance, see below). We do not count the ratio within total observations, as we derived it from reported observations. Records are a unique sampled area reported within a study. A study can provide multiple observations by reporting more than one variable, and/or by (for example) reporting variables from several distinct records, which could be locations or habitat types. The number of total observations in the database differs from that used in the statistical models because for species richness and seed abundance, the study needed to also report the area sampled and for seed density we removed zeros. We report more detail on that, and on other model sample sizes further down in the data section and under 'Model Specifications'.

Data	n	Number of observations used in each statistical model
Total number of studies checked from literature	7224	
Total number of studies with usable data	1442	
Total number of records	3096	
Total number of observations (richness, abundance and density combined)	8087	
Total species (richness)	2872	2792
Total seeds (abundance)	2585	2569
Number of seeds/m^2 (density)	2630	2626

Table S2. Details on each univariate hierarchical mixed effects model for main analysis. Poisson distributions with a log-link function were selected for models where the response was species richness. Lognormal distributions were selected for density and ratio models, with zeros removed (observations where zero seeds were found) as the log of zero is unknown. Bulk Effective Sample Sizes are reported. All models resulted in a Rhat of 1. Within a model short-name group, if the formula is blank, it follows the same formula as the first mentioned (tundra, top row of each model group) formulas are only listed after that diverge from that first listed formula.

Response	Formula	Biome	N observations	Distribution	Iterations	Effective Sample Size
Species richness	richness ~ centred log area * degraded * biome subtype + centred log number of sites + (1 | study / record)	Tundra	43	Poisson	2000	1202-2507
		Forest	734	Poisson	2000	1399-4424
		Grasslands	882	Poisson	2000	577-2240
		Mediterranean & Deserts	330	Poisson	8000	8895-24316
	richness ~ centred log area * biome subtype + centred log number of sites + (1 | study / record)	Arable	257	Poisson	6000	9156-19042
		Wetlands	472	Poisson	2000	1406-4129

Response	Formula	Biome	N observations	Distribution	Iterations	Effective Sample Size
	richness ~ centred log area * degraded + centred log number of sites + (1 | study / record)	Aquatic	73	Poisson	2000	942-2836
Seed density	seed density m2 ~ degraded * biome subtype + (1 | study / record)	Tundra	41	Lognormal	10000	17277-26499
		Forest	749	Lognormal	10000	7736-8703
		Grasslands	796	Lognormal	10000	7024-18898
		Mediterranean & Deserts	300	Lognormal	10000	13365-18766
	seed density m2 ~ biome subtype + (1 | study / record)	Arable	256	Lognormal	10000	14238-17036
		Wetlands	420	Lognormal	10000	9620-10391
	seed density m2 ~ degraded + (1 | study / record)	Aquatic	63	Lognormal	10000	10520-18130

Table S3. Richness-area slopes. Summary of model estimates regarding species richness slope as a function of area. In each column, the model estimate is followed by the lower credible interval and upper 50% credible interval, followed by 90% in brackets. Biomes and Realms presented in the same order as in Figures.

Realm	Biome	Ecoregion	Degraded	Slope and Intervals
Terrestrial	Tundra	Tundra	1	0.1063 (0.0405-0.1519 , 0.0073-0.266)
Terrestrial	Tundra	Tundra	0	0.2053 (0.135-0.265 , 0.0649-0.3773)
Terrestrial	Forest	Boreal	1	0.1018 (0.0803-0.1261 , 0.0384-0.152)
Terrestrial	Forest	Boreal	0	0.1163 (0.0936-0.1412 , 0.0507-0.172)
Terrestrial	Forest	Temperate	1	0.1494 (0.1328-0.1657 , 0.1077-0.1925)
Terrestrial	Forest	Temperate	0	0.1164 (0.0939-0.141 , 0.0517-0.1682)
Terrestrial	Forest	Tropical	1	0.1472 (0.1304-0.1636 , 0.1063-0.1873)
Terrestrial	Forest	Tropical	0	0.1136 (0.0919-0.1381 , 0.0491-0.1659)
Terrestrial	Grassland	Temperate and Boreal	1	0.0858 (0.0713-0.1004 , 0.0511-0.1202)
Terrestrial	Grassland	Temperate and Boreal	0	0.1215 (0.0987-0.1427 , 0.0733-0.1762)
Terrestrial	Grassland	Tropical	1	0.0997 (0.0843-0.1153 , 0.0616-0.138)
Terrestrial	Grassland	Tropical	0	0.1033 (0.0859-0.1204 , 0.0626-0.1448)
Terrestrial	Mediterranean and Desert	Deserts and Xeric Shrublands	1	0.148 (0.1272-0.1697 , 0.0942-0.1994)
Terrestrial	Mediterranean and Desert	Deserts and Xeric Shrublands	0	0.2129 (0.1708-0.2497 , 0.1269-0.3187)
Terrestrial	Mediterranean and Desert	Mediterranean Forests, Woodlands and Scrub	1	0.1667 (0.1455-0.1881 , 0.114-0.2191)
Terrestrial	Mediterranean and Desert	Mediterranean Forests, Woodlands and Scrub	0	0.1752 (0.1514-0.199 , 0.1152-0.2353)

Terrestrial	Arable	Mediterranean and Desert	1	0.0476 (0.0243-0.0672 , 0.0057-0.1008)
Terrestrial	Arable	Temperate and Boreal	1	0.0852 (0.0638-0.1054 , 0.0371-0.1357)
Terrestrial	Arable	Tropical	1	0.0865 (0.0572-0.1119 , 0.0268-0.1571)
Transitional	Wetland	Mediterranean and Desert	1	0.0766 (0.0443-0.1057 , 0.0117-0.149)
Transitional	Wetland	Mediterranean and Desert	0	0.1104 (0.0723-0.1449 , 0.0322-0.1998)
Transitional	Wetland	Temperate and Boreal	1	0.156 (0.133-0.1791 , 0.1-0.2115)
Transitional	Wetland	Temperate and Boreal	0	0.0936 (0.0603-0.1237 , 0.0245-0.1698)
Transitional	Wetland	Tropical	1	0.1574 (0.1177-0.1941 , 0.0683-0.252)
Transitional	Wetland	Tropical	0	0.1247 (0.0855-0.1595 , 0.0428-0.2206)
Aquatic	Aquatic	Aquatic	1	0.0801 (0.0298-0.1151 , 0.0062-0.2026)
Aquatic	Aquatic	Aquatic	0	0.131 (0.0753-0.1744 , 0.0329-0.2669)

Table S4. Average soil seed bank densities per realm. Realm mean, median and 50% and 90% upper and lower credible intervals based on the pooled fitted values of each model.

Realm	median	mean	50% Credible Interval	90% Credible Interval
Natural Terrestrial	3590	4214	3814-4565	3364-5217
Arable	11001	9434	8143-10508	6796-12733
Wetlands	10802	10318	8699-11626	7115-14481
Aquatic	9562	8124	5077-9867	3146-16700

Table S5. Summary of model estimates of seed density (m^{-2}). In each column, the model estimate (mean) is followed by the lower credible interval and upper 50% credible interval, followed by 90% in brackets. Biomes and Realms presented in the same order as in Figures.

Realm	Biome	Ecoregion	Degraded	Density and Intervals
Terrestrial	Tundra	Tundra	1	1262 15 (506-1491 , 242-3521)
Terrestrial	Tundra	Tundra	0	2000 15 (1415-2376 , 969-3580)
Terrestrial	Forest	Boreal	1	3381 14 (2183-4159 , 1375-6572)
Terrestrial	Forest	Boreal	0	1976 14 (1577-2292 , 1202-3001)
Terrestrial	Forest	Temperate	1	3590 13 (3214-3926 , 2782-4533)
Terrestrial	Forest	Temperate	0	2793 13 (2556-3007 , 2272-3385)
Terrestrial	Forest	Tropical	1	2061 12 (1838-2259 , 1586-2622)
Terrestrial	Forest	Tropical	0	1278 12 (1148-1395 , 999-1604)
Terrestrial	Grassland	Temperate and Boreal	1	6129 11 (5654-6564 , 5075-7324)
Terrestrial	Grassland	Temperate and Boreal	0	6737 11 (6286-7156 , 5731-7838)
Terrestrial	Grassland	Tropical	1	1755 10 (1383-2043 , 1046-2711)
Terrestrial	Grassland	Tropical	0	2844 10 (2326-3264 , 1833-4151)
Terrestrial	Mediterranean and Desert	Deserts and Xeric Shrublands	1	1760 8 (1395-2049 , 1055-2711)
Terrestrial	Mediterranean and Desert	Deserts and Xeric Shrublands	0	1975 8 (1656-2243 , 1339-2774)
Terrestrial	Mediterranean and Desert	Mediterranean Forests, Woodlands and Scrub	1	4567 9 (3827-5176 , 3085-6455)

Terrestrial	Mediterranean and Desert	Mediterranean Forests, Woodlands and Scrub	0	5170 9 (4463-5771 , 3689-6962)
Terrestrial	Arable	Mediterranean and Desert	1	11003 6 (8739-12770 , 6636-16829)
Terrestrial	Arable	Temperate and Boreal	1	11001 7 (9797-12051 , 8436-14047)
Terrestrial	Arable	Tropical	1	4200 5 (3391-4833 , 2633-6317)
Transitional	Wetland	Mediterranean and Desert	1	6544 3 (3349-8196 , 1735-15653)
Transitional	Wetland	Mediterranean and Desert	0	11845 3 (8377-14288 , 5760-21058)
Transitional	Wetland	Temperate and Boreal	1	11078 4 (9226-12588 , 7404-15767)
Transitional	Wetland	Temperate and Boreal	0	10802 4 (9487-11942 , 8036-14127)
Transitional	Wetland	Tropical	1	4400 2 (2688-5433 , 1638-9112)
Transitional	Wetland	Tropical	0	7042 2 (5422-8289 , 4001-11296)
Aquatic	Aquatic	Aquatic	1	4794 1 (2727-5898 , 1556-10664)
Aquatic	Aquatic	Aquatic	0	9562 1 (6092-11581 , 3833-19307)

Table S6. Predicted estimates of diversity at different scales. alpha = 0.01 m^2, gamma = 15 m^2. In the main results, we report the predicted alpha and gamma values for one site. Here, the number after each underscore specifies the predictions at each scale for the 3 levels of number of sites. Table sorted in ascending average alpha richness values at 1 site, average gamma richness values at 1 site and alphabetical order of Biomes.

Realm	Biome	Ecoregion	Degraded	0.01	1	15
Terrestrial	Tundra	Tundra	0	3 (1-4 , 0-7)	14 (17,11 , 7,23)	15 (7-19 , 3-37)
Terrestrial	Tundra	Tundra	1	3 (2-5 , 0-8)	11 (14,8 , 4,20)	8 (4-10 , 1-18)
Terrestrial	Forest	Boreal	0	10 (7-12 , 4-16)	20 (23,17 , 12,28)	21 (17-25 , 13-31)
Terrestrial	Forest	Boreal	1	10 (7-12 , 4-16)	20 (23,16 , 12,28)	20 (16-24 , 12-30)
Terrestrial	Forest	Temperate	0	11 (9-14 , 6-18)	30 (34,27 , 22,40)	40 (35-46 , 27-55)
Terrestrial	Forest	Temperate	1	12 (9-14 , 6-19)	29 (33,25 , 21,39)	36 (31-41 , 24-49)
Terrestrial	Forest	Tropical	0	13 (10-15 , 7-20)	34 (38,29 , 24,44)	44 (38-49 , 31-58)
Terrestrial	Forest	Tropical	1	13 (10-16 , 7-20)	32 (36,28 , 22,42)	39 (33-43 , 27-52)
Terrestrial	Grasslands	Temperate and Boreal	0	14 (11-16 , 8-21)	32 (35,28 , 23,41)	29 (25-34 , 19-40)
Terrestrial	Grasslands	Temperate and Boreal	1	14 (11-17 , 8-21)	31 (34,27 , 22,40)	26 (22-31 , 17-37)
Terrestrial	Grasslands	Tropical	0	14 (11-16 , 7-21)	35 (39,31 , 25,46)	36 (31-41 , 24-50)
Terrestrial	Grasslands	Tropical	1	14 (11-17 , 8-21)	33 (37,28 , 23,43)	30 (25-34 , 19-41)
Terrestrial	Mediterranean and Desert	Deserts and Xeric Shrublands	0	10 (8-13 , 5-17)	23 (27,20 , 15,32)	39 (32-44 , 25-54)
Terrestrial	Mediterranean and Desert	Deserts and Xeric Shrublands	1	11 (8-13 , 5-18)	22 (25,18 , 14,31)	32 (27-37 , 20-45)
Terrestrial	Mediterranean and Desert	Mediterranean Forests, Woodlands and Scrub	0	14 (11-17 , 7-22)	37 (41,33 , 27,48)	73 (63-83 , 50-99)

Terrestrial	Mediterranean and Desert	Mediterranean Forests, Woodlands and Scrub	1	15 (12-18 , 8-23)	32 (36,28 , 23,43)	51 (43-58 , 34-70)
Terrestrial	Arable	Mediterranean and Desert	1	13 (10-15 , 7-20)	25 (28,21 , 16,33)	18 (14-22 , 10-28)
Terrestrial	Arable	Temperate and Boreal	1	12 (9-14 , 6-19)	26 (30,23 , 18,36)	23 (18-27 , 13-34)
Terrestrial	Arable	Tropical	1	13 (10-16 , 6-21)	29 (33,25 , 20,40)	25 (20-29 , 15-38)
Transitional	Wetland	Mediterranean and Desert	0	11 (8-14 , 5-18)	22 (25,18 , 14,31)	21 (17-25 , 12-33)
Transitional	Wetland	Mediterranean and Desert	1	11 (8-14 , 5-19)	21 (24,17 , 13,30)	20 (15-24 , 11-31)
Transitional	Wetland	Temperate and Boreal	0	11 (8-13 , 5-17)	30 (33,26 , 21,39)	42 (34-48 , 26-60)
Transitional	Wetland	Temperate and Boreal	1	11 (8-13 , 5-18)	27 (31,23 , 18,37)	35 (28-40 , 21-51)
Transitional	Wetland	Tropical	0	9 (6-11 , 3-15)	26 (30,23 , 17,36)	42 (33-50 , 24-65)
Transitional	Wetland	Tropical	1	10 (7-12 , 4-17)	24 (27,20 , 15,33)	30 (24-36 , 17-48)
Aquatic	Aquatic	Aquatic	0	6 (3-8 , 1-12)	13 (16,10 , 7,22)	15 (9-20 , 4-33)
Aquatic	Aquatic	Aquatic	1	5 (3-6 , 1-11)	9 (12,7 , 4,16)	9 (5-12 , 2-20)

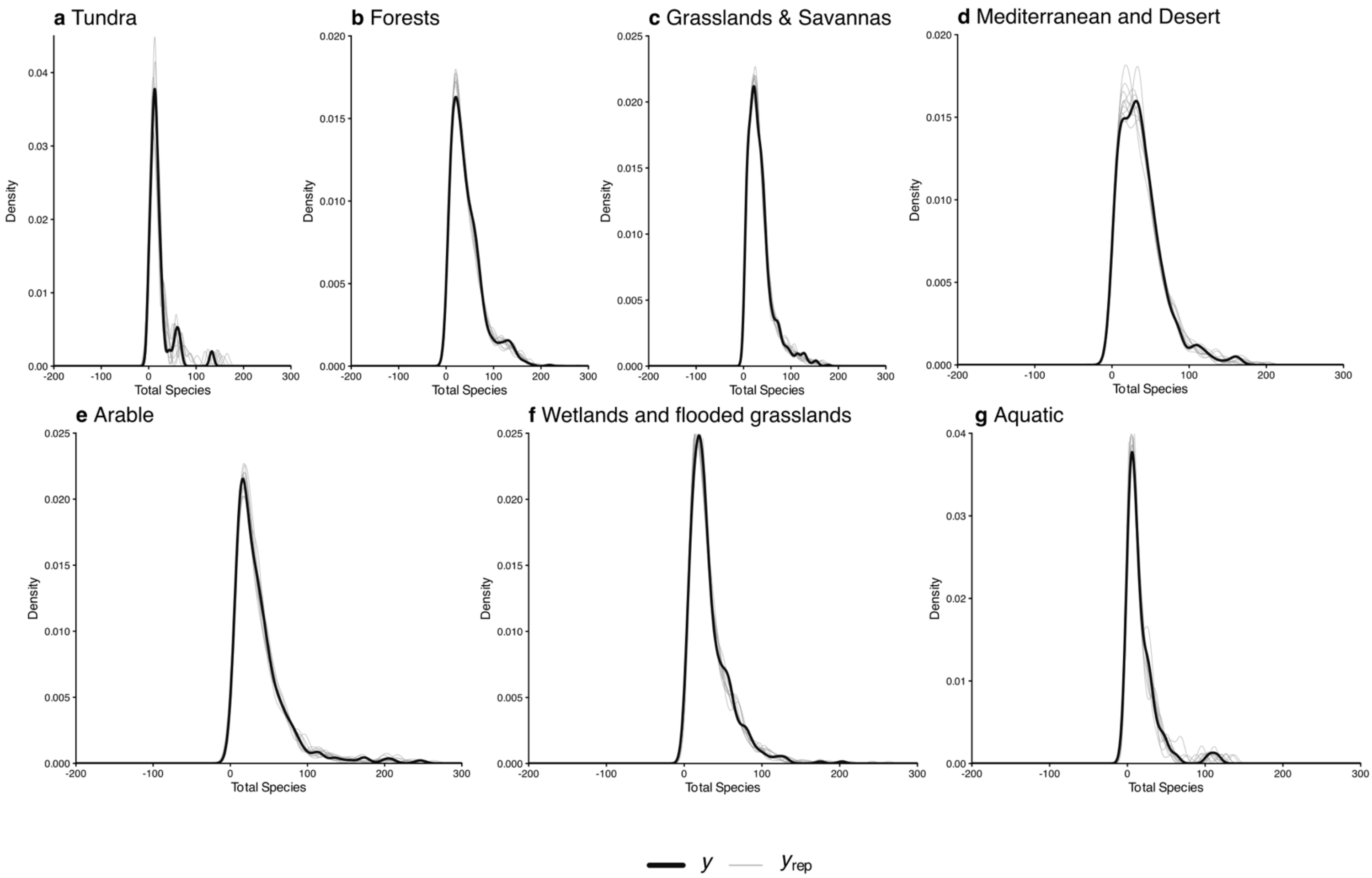

Figure S1. Model fit for species richness models. Predicted values for each species richness biome model (**a** Tundra, **b** Forests, **c** Grasslands and savannas, **d**, Mediterranean and desert, **e** Arable, **f** Wetlands and flooded grasslands, **g** Aquatic), indicated by the grey lines and observed values of the data indicated by the black line.

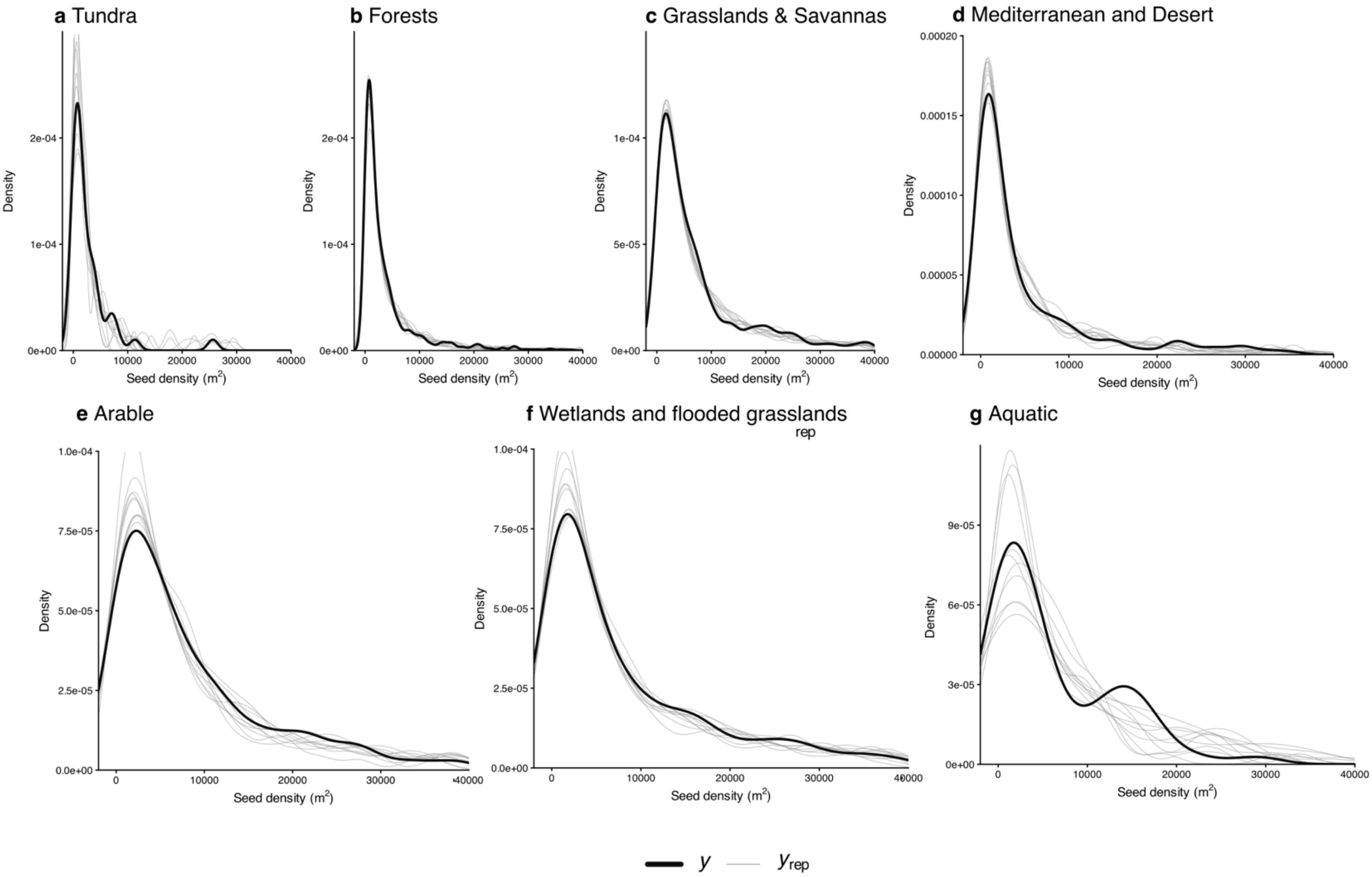

Figure S2. Model fit for seed density models. Predicted values for each seed density biome model (**a** Tundra, **b** Forests, **c** Grasslands and savannas, **d**, Mediterranean and desert, **e** Arable, **f** Wetlands and flooded grasslands, **g** Aquatic), indicated by the grey lines and observed values of the data indicated by the black line.

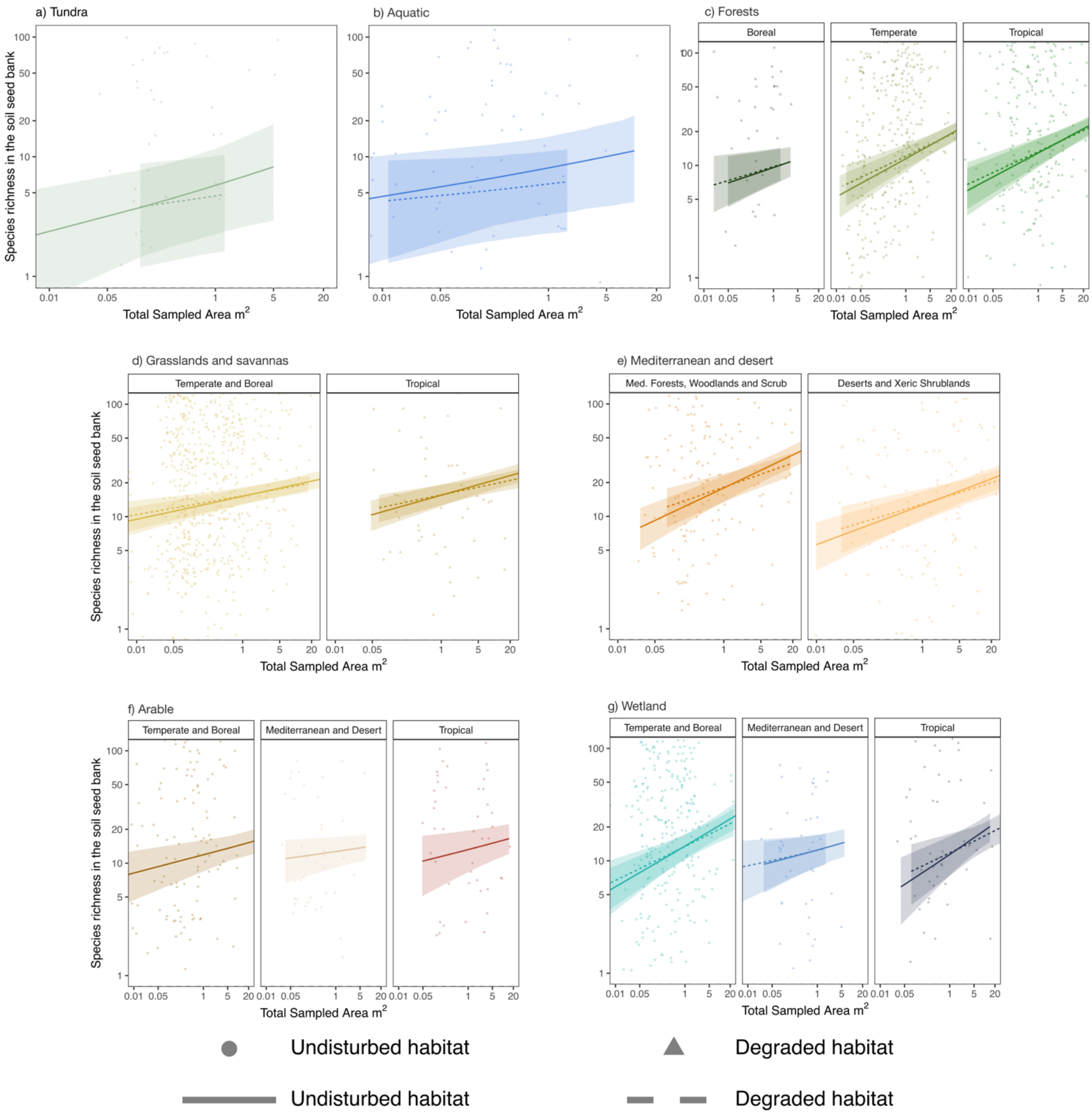

Figure S3. Species richness in the soil seed bank as a function of total soil samples (m^2). Each point and line type represents an undisturbed habitat (circles, solid line), and disturbed habitat (triangles, dotted line). Each point is the species richness reported for a given total area of soil sampled in the literature, each line is the overall estimated relationship between the number of species and sampled area for one site, and the shaded area around lines are the 95% credible intervals. Panels represent biomes (**a** Tundra, **b** Aquatic, **c** Forests, **d** Grasslands and savannas, **e** Mediterranean and desert, **f** Arable, **g** Wetlands and flooded grasslands,), with subpanels representing ecosystems. Sample sizes, slope estimates and credible intervals can be found in Tables 1 & S3.
