# Supplementary material for: Divergent patterns of richness and density in the global soil seed bank
Source: Nat Commun. 2026 Aug 22;17:8820. doi: 10.1038/s41467-026-77079-x (PMC13499811; doi:10.1038/s41467-026-77079-x)
Supplement: Supplementary file 2 [file 41467_2026_77079_MOESM2_ESM.pdf]

nature portfolio

Corresponding author(s): Alistair Auffret, Emma Ladouceur

Last updated by author(s): Jul 7, 2026

Reporting Summary

Nature Portfolio wishes to improve the reproducibility of the work that we publish. This form provides structure for consistency and transparency in reporting. For further information on Nature Portfolio policies, see our Editorial Policies and the Editorial Policy Checklist.

Statistics

For all statistical analyses, confirm that the following items are present in the figure legend, table legend, main text, or Methods section.

n/a	Confirmed	
☐	☒	The exact sample size (*n*) for each experimental group/condition, given as a discrete number and unit of measurement
☐	☒	A statement on whether measurements were taken from distinct samples or whether the same sample was measured repeatedly
☐	☒	The statistical test(s) used AND whether they are one- or two-sided<br>*Only common tests should be described solely by name; describe more complex techniques in the Methods section.*
☐	☒	A description of all covariates tested
☐	☒	A description of any assumptions or corrections, such as tests of normality and adjustment for multiple comparisons
☐	☒	A full description of the statistical parameters including central tendency (e.g. means) or other basic estimates (e.g. regression coefficient) AND variation (e.g. standard deviation) or associated estimates of uncertainty (e.g. confidence intervals)
☒	☐	For null hypothesis testing, the test statistic (e.g. *F*, *t*, *r*) with confidence intervals, effect sizes, degrees of freedom and *P* value noted<br>*Give P values as exact values whenever suitable.*
☐	☒	For Bayesian analysis, information on the choice of priors and Markov chain Monte Carlo settings
☐	☒	For hierarchical and complex designs, identification of the appropriate level for tests and full reporting of outcomes
☒	☐	Estimates of effect sizes (e.g. Cohen's *d*, Pearson's *r*), indicating how they were calculated

Our web collection on statistics for biologists contains articles on many of the points above.

Software and code

Policy information about availability of computer code

Data collection	No software was used for data collection.
Data analysis	Data were analysed using the open-source software R Project for Statistical Computing version 4.4.0. Packages used for data handling and analysis were brms version 2.23.0, tidyverse version 2.0.0, bayesplot version 1.15.0 and ggplot2 version 4.0.3. All software is fully cited in the reference list. The code used to reproduce the analyses is hosted at the Github repository https://github.com/Global-Seed-Banks/Seed-Banks, with the version used for analysis archived at Zenodo (https://doi.org/10.5281/zenodo.21008573).

For manuscripts utilizing custom algorithms or software that are central to the research but not yet described in published literature, software must be made available to editors and reviewers. We strongly encourage code deposition in a community repository (e.g. GitHub). See the Nature Portfolio guidelines for submitting code & software for further information.

Data

Policy information about availability of data

All manuscripts must include a data availability statement. This statement should provide the following information, where applicable:

- Accession codes, unique identifiers, or web links for publicly available datasets
- A description of any restrictions on data availability
- For clinical datasets or third party data, please ensure that the statement adheres to our policy

The full dataset used for analysis – The global seed bank database – is deposited at the Swedish National Data Service at https://doi.org/10.5878/bvs7-gk47 103.

Methods for the literature search, data extraction, and citations of all 1442 component studies can be found in Auffret et al. (2024; https://doi.org/10.1002/ecy.4438). Modelled values relating to the box-and-whiskers of Figures 2-4 are provided in the Tables S3-S5, while model objects and scripts needed to reproduce the plots are available from the above-referenced code repository.

Research involving human participants, their data, or biological material

Policy information about studies with human participants or human data. See also policy information about sex, gender (identity/presentation), and sexual orientation and race, ethnicity and racism.

Reporting on sex and gender	N/A
Reporting on race, ethnicity, or other socially relevant groupings	N/A
Population characteristics	N/A
Recruitment	N/A
Ethics oversight	N/A

Note that full information on the approval of the study protocol must also be provided in the manuscript.

Field-specific reporting

Please select the one below that is the best fit for your research. If you are not sure, read the appropriate sections before making your selection.

☐ Life sciences ☐ Behavioural & social sciences ☒ Ecological, evolutionary & environmental sciences

For a reference copy of the document with all sections, see nature.com/documents/nr-reporting-summary-flat.pdf

Ecological, evolutionary & environmental sciences study design

All studies must disclose on these points even when the disclosure is negative.

Study description

Here follows a brief description of data collection. Please see the published data descriptor for full details (https://doi.org/10.1002/ecy.4438).
We conducted a literature search of soil seed bank published peer reviewed literature. We collected information regarding the richness (number of species), abundance (number of seeds) and density (number of seeds per unit area), as well as sampling information, including geographic location and description of the habitats in which the seed bank was sampled.

Research sample

Our sample includes a complete list of all soil seed bank studies included in the Web of Science as of 31 December 2019, as well as studies from a Russian-language database. Data included were those papers where the relevant response variables and adequate sampling information were present in the published paper.

Sampling strategy

Web of Science search
We searched the Web of Science database (https://www.webofknowledge.com) with the term seedbank* OR "seed bank*" in the topic field, including articles from the years 1945-2019. This search returned 7536 results, for which bibliographic information was exported and subsequently collated into one file. This file was then filtered using the indexing functions in the R statistical environment (R Core Team 2022), to only include Journal articles (entries of J in the exported file column PT), resulting in 7106 studies to be examined.

Additional studies
During data entry, a small number of relevant studies were identified that had not been captured in the Web of Science search, and were added to the database. These were mostly older studies that were carried out prior to the widespread use of the term 'seed bank'.

Russian database search
During the course of the data collection, it became apparent that there was an especially large geographical data gap in and around the Russian Federation. We therefore added publications from an existing database of seed bank studies from the former USSR containing studies from 1965 to 2004, compiled by collaborator Vladimir Onipchenko. This database was then complemented with a literature search on the Russian language academic database at http://www.elibrary.ru, to find more recent data sources. The search terms used were: "ЖИЗНЕСПОСОБНЫЕ СЕМЕНА В ПОЧВЕ" (49 results; approximately: viable seeds in soil), "ПОЧВЕННЫЙ БАНК СЕМЯН" (39 results; soil seed bank), "ЗАПАС СЕМЯН В ПОЧВЕ" (281 results; seed stock in soil). The same search terms were used in Google Scholar (https://scholar.google.com), and any other relevant studies identified in reference lists were consulted.

Data collection

Studies of the full seed bank community were then checked, and information relating to the richness, abundance and density of seeds in the soil were extracted where available

Timing and spatial scale	Studies included in the database were published from 1940-2020.
Data exclusions	No data that were included in the published database were excluded from our analysis.
Reproducibility	We published all data open access and will publish all code to reproduce results open access.
Randomization	All published studies in the peer reviewed literature that we used were randomized.
Blinding	Not applicable to out approach.

Did the study involve field work? ☐ Yes ☒ No

Reporting for specific materials, systems and methods

We require information from authors about some types of materials, experimental systems and methods used in many studies. Here, indicate whether each material, system or method listed is relevant to your study. If you are not sure if a list item applies to your research, read the appropriate section before selecting a response.

Materials & experimental systems

n/a	Involved in the study
☒	☐ Antibodies
☒	☐ Eukaryotic cell lines
☒	☐ Palaeontology and archaeology
☒	☐ Animals and other organisms
☒	☐ Clinical data
☒	☐ Dual use research of concern
☒	☐ Plants

Methods

n/a	Involved in the study
☒	☐ ChIP-seq
☒	☐ Flow cytometry
☒	☐ MRI-based neuroimaging

Plants

Seed stocks	*Report on the source of all seed stocks or other plant material used. If applicable, state the seed stock centre and catalogue number. If plant specimens were collected from the field, describe the collection location, date and sampling procedures.*
Novel plant genotypes	*Describe the methods by which all novel plant genotypes were produced. This includes those generated by transgenic approaches, gene editing, chemical/radiation-based mutagenesis and hybridization. For transgenic lines, describe the transformation method, the number of independent lines analyzed and the generation upon which experiments were performed. For gene-edited lines, describe the editor used, the endogenous sequence targeted for editing, the targeting guide RNA sequence (if applicable) and how the editor was applied.*
Authentication	*Describe any authentication procedures for each seed stock used or novel genotype generated. Describe any experiments used to assess the effect of a mutation and, where applicable, how potential secondary effects (e.g. second site T-DNA insertions, mosiacism, off-target gene editing) were examined.*
