# Supplementary material for: Divergent patterns of richness and density in the global soil seed bank
Source: Nat Commun. 2026 Aug 22;17:8820. doi: 10.1038/s41467-026-77079-x (PMC13499811; doi:10.1038/s41467-026-77079-x)
Supplement: Supplementary file 3 [file 41467_2026_77079_MOESM3_ESM.pdf]

nature portfolio

Peer Review File

Divergent patterns of richness and density in the global soil seed bank

Corresponding Author: Dr Alistair Auffret

This file contains all reviewer reports in order by version, followed by all author rebuttals in order by version.

Version 0:

Reviewer comments:

Reviewer #1

(Remarks to the Author)
Leveraging a global database spanning all seven continents, the authors investigated spatial patterns in soil seed banks by analyzing species richness and seed density. They examined multiple components of soil seed bank diversity—such as species richness, abundance, and seed density—at two spatial resolutions. Their analysis compared diversity both within and across broad habitat categories and biomes, and assessed the effects of habitat degradation on seed banks. The authors revealed several compelling large-scale patterns in soil seed banks, advancing our understanding of seed bank ecology and its implications for global biodiversity distribution and dynamics. Up to now, such large-scale analyses are still quite lacking in the field of soil seed bank ecology.
Despite these strengths, I believe the manuscript still has certain limitations, as outlined below.
1. While the authors have identified several patterns from the global dataset, the manuscript lacks an exploration of the underlying mechanisms. The content, particularly in the Results and Discussion sections, remains largely descriptive.
2.Although the authors emphasize that soil seed banks play a key role in shaping global biodiversity patterns across biomes, the presented results provide limited explanatory power for understanding biodiversity at a global scale.
3.The relationship between species richness and seed density patterns does not, in itself, constitute a novel or compelling research question.
4. The study reports higher soil seed bank richness in undisturbed versus degraded habitats, but the criteria for defining "degraded habitat" are unclear. A consistent, quantitative standard applied across all global study sites is lacking. For instance, seed bank richness and density might initially increase in slightly degraded habitats but decrease in severely degraded ones compared to undisturbed conditions. This non-linear response warrants a more nuanced classification of habitat degradation.
5.The study appears somewhat non-hypothesis-driven. The current structure—Introduction (6 paragraphs), Methods (4 parts), Results (5 parts), and Discussion (4 parts)—lacks a consistent and logical flow. I recommend restructuring each section around the core scientific questions or specific research topics of this study to enhance clarity and coherence.
Specific comments and suggestions
For Introduction
It would be beneficial for the authors to incorporate clear research questions or hypotheses in the Introduction. This would provide a stronger logical framework and enhance the focus of the entire manuscript, particularly in the Results and Discussion sections.
Line 73.The term "abundance" is ambiguous—does it refer to the number of species (richness) or the quantity of seeds? The key metrics of "species richness," "abundance," and "seed density" should be clearly defined in the Methods section.
Line 71-75. In the final sentence of the abstract's first paragraph, the authors mention the effect of warming/temperature change on the seed bank. However, this topic is not addressed in the subsequent analyses or results of the manuscript. I recommend revising the conclusion of the abstract to align with the study's actual focus.
For Method
Line 143-152. The content of this paragraph doesn’t looks like a method.
How to define the degraded habitat for all the data from different habitats? What is the standard of degraded habitat in each site of the global data?
For Results
Line 314. I suggest change the title ‘Soil seed banks of the world’, and make it more focus on the question.
Line 315-316. It is not clear about the meaning of 35932626, is it total seed numbers in the global study? Why you show this number (similarly hereinafter)? It has no substantive meaning because it will change with you collected data size change.

Line 323-324. 3 to 501119 seeds m-2, this is an extremely huge difference in forest ecosystem. It fully demonstrates the significant differences in your collected data.
For Discussion
The structure of Discussion (four parts) was not based on the scientific questions of this manuscript, also not keep consist with Method and Results sections. Especially the third (global inghts…) and four part (the growth….).
Line 481-508 (Scale-dependence of species richness in soil seed banks worldwide). This paragraph is very descriptive
Line 510-535. The topic of this part is patterns and determinants of seed bank and seed density, only patterns here, but few determinants here.
Line 537-553. Returning to the issue mentioned earlier, how to define degradation and how to determine the degree of degradation - these factors largely determine the result, namely the changes in species diversity and seed density. Moreover, this paragraph contains descriptive and speculative content.
Line 555-574 (Global insights into seed bank sampling). I'm not sure whether this topic is necessary to discuss in this Discussion section.

(Remarks on code availability)

Reviewer #2

(Remarks to the Author)
The manuscript presents a comprehensive global analysis of soil seed banks, integrating over 1,400 studies across all continents. The findings highlight important divergences between species richness and seed density, the impact of habitat degradation on seed bank richness, and clear contrasts among biomes. The study is original, relevant, and provides a valuable global synthesis that advances our understanding of biodiversity and ecological restoration. The methodology is appropriate and clearly described, ensuring reproducibility. Nevertheless, the manuscript would benefit from a more detailed explanation of how differences in sampling effort across studies were handled, as well as a clearer discussion of potential sampling biases (e.g., overrepresentation of agricultural and temperate ecosystems). In addition, the authors could further emphasize the effects of heterogeneity in sampling scales and regional coverage.
Recommendation
I recommend publication after minor revision, mainly to clarify potential biases, strengthen the discussion of uncertainty, and ensure figure and table legends are fully self-explanatory.

(Remarks on code availability)

Reviewer #3

(Remarks to the Author)
I co-reviewed this manuscript with one of the reviewers who provided the listed reports. This is part of the Nature Communications initiative to facilitate training in peer review and to provide appropriate recognition for Early Career Researchers who co-review manuscripts.

(Remarks on code availability)

Version 1:

Reviewer comments:

Reviewer #2

(Remarks to the Author)
The revised version of the manuscript "Divergent patterns of richness and density in the global soil seed bank", together with the authors' detailed response to the reviewers' comments, has been carefully examined.

All major and minor points raised during the review process have been adequately addressed. In particular, the manuscript now shows clear improvements in the interpretation and discussion of the results, with a more explicit integration of biogeographical patterns, disturbance regimes, and plant regeneration strategies. The scope and wording of the Abstract have been appropriately refined to better reflect the core findings and to avoid overgeneralisation.

The definition and treatment of habitat degradation have been clarified in the Methods and critically discussed in the Discussion, including an explicit acknowledgement of the limitations associated with the binary classification used. Potential biases related to heterogeneous sampling effort and data availability across ecosystems have also been more thoroughly addressed, and the revised text provides a transparent and balanced assessment of the robustness of the global patterns identified.

In addition, the overall structure and clarity of the manuscript have been improved, and the figures and tables are now clear, informative, and consistent with the journal's standards.

Based on this final assessment, the manuscript meets the scientific and editorial standards of the journal, and no further revisions are required from the perspective of this reviewer.

(Remarks on code availability)
The code and associated materials were reviewed. The documentation provides sufficient information to assess the methodology and supports the reproducibility of the results. However, the code was not executed as part of this review.

Reviewer #3

(Remarks to the Author)
I co-reviewed this manuscript with one of the reviewers who provided the listed reports. This is part of the Nature Communications initiative to facilitate training in peer review and to provide appropriate recognition for Early Career Researchers who co-review manuscripts.

(Remarks on code availability)
The code and associated materials were reviewed. The documentation provides sufficient information to assess the methodology and supports the reproducibility of the results. However, the code was not executed as part of this review.

Open Access This Peer Review File is licensed under a Creative Commons Attribution 4.0 International License, which permits use, sharing, adaptation, distribution and reproduction in any medium or format, as long as you give appropriate credit to the original author(s) and the source, provide a link to the Creative Commons license, and indicate if changes were made.
In cases where reviewers are anonymous, credit should be given to 'Anonymous Referee' and the source.
The images or other third party material in this Peer Review File are included in the article's Creative Commons license, unless indicated otherwise in a credit line to the material. If material is not included in the article's Creative Commons license and your intended use is not permitted by statutory regulation or exceeds the permitted use, you will need to obtain permission directly from the copyright holder.
To view a copy of this license, visit https://creativecommons.org/licenses/by/4.0/

Divergent patterns of richness and density in the global soil seed bank

Response to referees.

Dear reviewers,

Many thanks for considering this new and improved version of our manuscript. We have worked hard to improve the logical flow of the manuscript and have included a deeper exploration of the mechanisms behind the patterns that we identified from our analysis. We have also improved the figures and the manuscript text for clarity. Please see our responses to the referees' individual comments below, and the revised manuscript, which is now formatted according to journal guidelines.

Best wishes,
Alistair Auffret and co-authors.

REVIEWER COMMENTS

Reviewer #1 (Remarks to the Author):

Leveraging a global database spanning all seven continents, the authors investigated spatial patterns in soil seed banks by analyzing species richness and seed density. They examined multiple components of soil seed bank diversity—such as species richness, abundance, and seed density—at two spatial resolutions. Their analysis compared diversity both within and across broad habitat categories and biomes, and assessed the effects of habitat degradation on seed banks. The authors revealed several compelling large-scale patterns in soil seed banks, advancing our understanding of seed bank ecology and its implications for global biodiversity distribution and dynamics. Up to now, such large-scale analyses are still quite lacking in the field of soil seed bank ecology.
Despite these strengths, I believe the manuscript still has certain limitations, as outlined below.
Many thanks for your review of our work. We have taken serious consideration of your comments and improved our manuscript accordingly. Please see our responses to your specific comments below.

1. While the authors have identified several patterns from the global dataset, the manuscript lacks an exploration of the underlying mechanisms. The content, particularly in the Results and Discussion sections, remains largely descriptive.
Thank you for this comment. Although we retained the character of our Results section, where we focused on describing our findings, we made wholesale changes to the Discussion. In addition to rearranging the Discussion in order to create a more logical flow throughout the paper (in response to a later comment), we carefully edited all of the main paragraphs. Now, rather than only broadly describing our results and relating them to previous findings, we describe in much more detail how the latitudinal biodiversity gradient and the disturbance regimes that exist in different ecosystems combine with the regeneration strategies of the plant communities found in that environment to determine our observed patterns in the richness and density of the soil seed bank . By linking our findings to the ecology of the systems in question like this, we think that these changes result in a much stronger manuscript.

2.Although the authors emphasize that soil seed banks play a key role in shaping global biodiversity patterns across biomes, the presented results provide limited explanatory power for understanding biodiversity at a global scale.
We understand that the final clause of the Abstract in our submitted manuscript "with implications for understanding biodiversity at the global scale." was potentially somewhat of an exaggeration. We have toned this down in the new version to "give valuable insights to the understanding of plant biodiversity worldwide." (Lines 51-53). We think that this statement is reasonable, being more conservative and focusing on plants, and also that the statement is supported by our results. To list some examples, our results in relation to habitat degradation have not been shown before at this scale in the field of seed bank ecology, and the need to consider spatial grain when comparing seed bank richness across regions is another important finding. Finally, we think that the broad comparisons of richness and density in different ecosystems, and the revelation of divergent patterns in their relationship, is – despite being explained from previous ecological knowledge derived at smaller scales – important for understanding biogeographical and macroecological variation in seed bank diversity, and as a consequence plant ecology in general. We hope that the new version of the manuscript is more convincing in this respect, although the short word limit allowed by the journal means that it was not possible to add these specific points to the Abstract.

3.The relationship between species richness and seed density patterns does not, in itself, constitute a novel or compelling research question.
Thank you for this comment, but we respectfully disagree. The relationship between density and abundance (in our case transformed to density) in ecological assemblages is one of the classic questions in ecology. For example, the paper by Preston (1962, Ecology, https://doi.org/10.2307/1931976) is one of the papers in the volume *Foundations of Ecology* (1991, Eds: Real & Brown), and the issue is still producing interesting and important papers, for example Storch et al. (2018, Ecology Letters, https://doi.org/10.1111/ele.12941) and

Blowes et al. (2022, Ecology, https://doi.org/10.1002/ecy.3820). We think that our findings, and particularly the differences in patterns of richness and density across global biomes, ecosystems, and in response to habitat degradation, are an important addition to this topic. We are happy that Referee 2 agrees with this standpoint, writing “The findings highlight important divergences between species richness and seed density, the impact of habitat degradation on seed bank richness, and clear contrasts among biomes.”

4. The study reports higher soil seed bank richness in undisturbed versus degraded habitats, but the criteria for defining "degraded habitat" are unclear. A consistent, quantitative standard applied across all global study sites is lacking. For instance, seed bank richness and density might initially increase in slightly degraded habitats but decrease in severely degraded ones compared to undisturbed conditions. This non-linear response warrants a more nuanced classification of habitat degradation.

Thank you for highlighting this important point. Degradation was by necessity a binary classification based on the site descriptions by the authors of the component studies. Even if it were feasible for us to revisit the 1442 studies making up the database, we do not think that it would be possible to categorise the degraded sites based on any consistent quantitative classification, because the same level of information relating to the state and history of the site would not be consistently provided in the published papers.

We think that it is relevant and defensible to use a binary classification, and point to important global studies where binary or otherwise similarly broad classifications of (for example) habitat degradation are used. For example, our classification is arguably more detailed than that of the PREDICTS database, that has been used to investigate the global impacts of land-use change, and compares biodiversity primary vegetation to different types of degraded vegetation (whereas we compare undisturbed and degraded habitat separately per ecosystem), for example Newbold et al. (2020, Nature Ecol. Evol. https://doi.org/10.1038/s41559-020-01303-0). In other key papers, Atkinson et al. (2024, Ecology Letters, https://doi.org/10.1111/ele.14025) compare biodiversity outcomes in restored vs. reference sites, while Gray et al. (2016, Nature Communications, https://doi.org/10.1038/ncomms12306), among many others, compare biodiversity inside and outside of protected areas, while Vilà et al. (2016, Ecology Letters, https://doi.org/10.1111/j.1461-0248.2011.01628.x) compare communities containing or not containing invasive species.

Nonetheless, we do agree that changes were needed in our manuscript. First, in the Methods, we now give a much more detailed description of how habitat degradation was defined in this study (Lines 576-591). Second, we bring up the issue in the Discussion (Lines 450-467), remarking on how the large variation in what constitutes a degraded site can have affected our results, including your point regarding initial biodiversity increases in degraded systems.

5.The study appears somewhat non-hypothesis-driven. The current structure—Introduction (6 paragraphs), Methods (4 parts), Results (5 parts), and Discussion (4 parts)—lacks a consistent and logical flow. I recommend restructuring each section around the core scientific questions or specific research topics of this study to enhance clarity and coherence.

In rearranging our manuscript to fit the format of Nature Communications (i.e. Methods last), we have tried to restructure the paper to give it a clearer flow, and describe this below.

In the Introduction, we now put more focus on the potential differences in seed bank richness and density, which was not really covered before, but is our main result. In two paragraphs, we more explicitly mention these two components of the seed bank when talking about seed banking more broadly (Lines 66-81, 83-102) and introduces the background to our main findings more clearly. We have not added any specific hypotheses for the study, but, we did have a clear aim of what we wanted to investigate when putting together the global database. We think that the improvements made to the Introduction now contribute to a clear logical flow regarding why we should study global patterns in seed bank richness and density in the context of differences across regions, habitats and responses to disturbance. The Introduction now reads as follows: [1] Seed banks and biodiversity, [2] Differences across biomes, [3] Differences across habitats and with degradation, [4] Effect of spatial scale, [5-6] Summary of study focus and findings (communicating broad findings in the final paragraph of the Introduction is required by journal).

In the Results section, the subheadings now follow the order and content of the main research foci listed in the final paragraph of the Introduction. That is, we start with richness (Line 188-210), then move onto density (Lines 212-220). We then describe the results relating to habitat degradation (Lines 258-270), before finishing off with the synthetic element and our findings of divergent patterns of richness and density across our results (Lines 272-291).

In the Discussion, we altered the arrangement of paragraphs (please note that Nature Communications does not allow subheadings in the Discussion section) so that it now broadly reflects the results section. However, we found it necessary to diverge somewhat. As reflected in the title and abstract, we consider our main result to be the divergent patterns of richness and density. Because this is a result that emerges from our results as a whole, rather than from any specific model(s), it was the last subheading of our Results section. In the Discussion we discuss it in the same section as we discuss the richness and density results (Lines 347-381). There is also a section on the effects of habitat degradation

(Lines 383-400), spatial scaling effects (present in the original manuscript but improved and moved down to Lines 402-424), plus new versions of the previous manuscripts subsections on sampling (now altered to concentrate more on biases in our study, Lines 426-448), and a concluding section placing our study in the broad context of the literature (Lines 469-685). Therefore, the Discussion now has the same general structure as the research foci mentioned in the Introduction and the Results section, as well as having an overall structure broadly representative of the ecological literature. That is: [1] Summary of most important results and implications; [2] soil seed bank richness, density and diverging patterns; [3] habitat degradation; [4] spatial scale; [5] biases and caveats; [6] conclusions.

Specific comments and suggestions

For Introduction

It would be beneficial for the authors to incorporate clear research questions or hypotheses in the Introduction. This would provide a stronger logical framework and enhance the focus of the entire manuscript, particularly in the Results and Discussion sections.

As mentioned in detail above, we did not explicitly add hypotheses to the Introduction, but improved the whole section so that it feeds into the clear focal points of our study, which then form the basis of the structure of the Results and Discussion. Although it would be possible to re-formulate these focal points into questions, we found it difficult given the journal style of summarising the findings of the study at the end of the introduction, which would then result in us asking and then immediately answering the questions, which did not appeal to us stylistically (Lines 135-146).

Line 73.The term "abundance" is ambiguous—does it refer to the number of species (richness) or the quantity of seeds? The key metrics of "species richness," "abundance," and "seed density" should be clearly defined in the Methods section.

Thank you for pointing out this lack of clarity. We do not use abundance data (the total number of seeds from a given plot or set of plots described in a study) from the seed bank database in this paper, only density (which is abundance divided by unit area) and richness. To prevent confusion, we therefore only mention abundance in the part of the Methods section where the database is described (Line 490), and where richness, abundance and density are all defined (Line 496), and in the Discussion when describing richness-abundance relationships more broadly (Line 349). In relation to the comment below, the sentence highlighted here was removed from the Introduction. In a later point, we changed ‘abundant’ to ‘large’, when describing weed communities in arable fields (Line 93). We also removed the column for ‘species number’ in Table 1, as we do not analyse those data here.

Line 71-75. In the final sentence of the abstract's first paragraph, the authors mention the effect of warming/temperature change on the seed bank. However, this topic is not

addressed in the subsequent analyses or results of the manuscript. I recommend revising the conclusion of the abstract to align with the study's actual focus.
This sentence (in the introduction), was written to put seed banks in the context of the current biodiversity crisis. However, when re-reading the paragraph, we agree that mentioning time-lagged responses to land-use change, and expected impacts of climate change do not really fit here. Habitat degradation in the broad sense is important to the story (and introduced in a later paragraph), but climate change is less relevant. We have removed these sentences, and incorporated the point regarding land-use change into the discussion (Lines 459-461).

For Method
Line 143-152. The content of this paragraph doesn't looks like a method.
We agree. On re-reading the paragraph, we found it quite self-indulgent and didn't give any essential information, and we have therefore removed it.

How to define the degraded habitat for all the data from different habitats? What is the standard of degraded habitat in each site of the global data?
Please see our response to the earlier comment where we address this important point.

For Results
Line 314. I suggest change the title 'Soil seed banks of the world', and make it more focus on the question.
Line 315-316. It is not clear about the meaning of 35932626, is it total seed numbers in the global study? Why you show this number (similarly hereinafter)? It has no substantive meaning because it will change with you collected data size change.
Line 323-324. 3 to 501119 seeds m-2, this is an extremely huge difference in forest ecosystem. It fully demonstrates the significant differences in your collected data.
Thank you for raising these issues, which we address together. With this section, we thought it would be interesting to the reader to show the extent of the database, and that it would provide a few interesting insights into what kind of information the database contains, and at the same time honour the huge amount of effort that the global seed bank ecology community has put into measuring seed banks. By sharing the variation in the extent and findings of the studies, we also think that this adds important transparency to our study, as indicated by these comments and the comments of Referee 2 who also requested more information on biases in the data set. In rearranging the manuscript to fit the journal's required structure, we think that we found a good solution. Giving this section a new subheading (Summary information and data variation in the global seed bank) and moving it to the part of the Methods where the database is described, we are able to retain this important and interesting information, without disrupting the flow of the main text, where we agree that this section could be confusing or distracting (Lines 517-542).

For Discussion

The structure of Discussion (four parts) was not based on the scientific questions of this manuscript, also not keep consist with Method and Results sections. Especially the third (global inghts…) and four part (the growth….).

As addressed in detail in response to the earlier main comment, we rearranged our Discussion to ensure a more logical thread throughout the paper.

Line 481-508 (Scale-dependence of species richness in soil seed banks worldwide). This paragraph is very descriptive

We have now improved this paragraph. We do not go as far into depth into seed bank ecology here, compared to the new versions of other paragraphs in the discussion, because here we describe spatial and biogeographical trend in our results, and we think that it is beyond our scope to describe and explain for example the latitudinal biodiversity gradient and island biogeography in any great detail. Nonetheless, we have improved the paragraph to more clearly demonstrate the implications of our findings within this broad topic (Lines 402-424).

Line 510-535. The topic of this part is patterns and determinants of seed bank and seed density, only patterns here, but few determinants here.

Thank you. This comment is related to the main comment regarding the mechanisms behind our results. We think that the new version of the Discussion is much stronger, both in putting our results into context and explaining the patterns and processes behind the findings.

Line 537-553. Returning to the issue mentioned earlier, how to define degradation and how to determine the degree of degradation - these factors largely determine the result, namely the changes in species diversity and seed density. Moreover, this paragraph contains descriptive and speculative content.

As described earlier, we now describe the definition of degradation much more clearly. Here in the Discussion, we now discuss potential issues with the binary classification used, and how it may have affected our results (Lines 383-400). Also in relation to another comment, this was one of the paragraphs that we have put a lot of effort into improving, in terms of the ecology used to explain and contextualise our results.

Line 555-574 (Global insights into seed bank sampling). I'm not sure whether this topic is necessary to discuss in this Discussion section.

We have chosen to keep this paragraph to conclude our manuscript. We think that this is an excellent opportunity to give a broad overview of large-scale seed bank ecology, including sub-topics that are not otherwise linked to our study (Lines 469-485).

Reviewer #2 (Remarks to the Author):

The manuscript presents a comprehensive global analysis of soil seed banks, integrating over 1,400 studies across all continents. The findings highlight important divergences between species richness and seed density, the impact of habitat degradation on seed bank richness, and clear contrasts among biomes. The study is original, relevant, and provides a valuable global synthesis that advances our understanding of biodiversity and ecological restoration.

Thank you for your comments and positive reflections on our manuscript. We respond to your main comment regarding data bias and its effects on our results below.

The methodology is appropriate and clearly described, ensuring reproducibility. Nevertheless, the manuscript would benefit from a more detailed explanation of how differences in sampling effort across studies were handled, as well as a clearer discussion of potential sampling biases (e.g., overrepresentation of agricultural and temperate ecosystems). In addition, the authors could further emphasize the effects of heterogeneity in sampling scales and regional coverage.

Thank you for pointing this out so that our approaches, and their limitations, can be clearer for readers. In order to account for differences in sampling effort, across studies, we allowed our estimates for species richness to vary according to sampling effort, expressed as the total area of all soil core samples that went into each richness estimate coming from the literature. Additionally, because we used hierarchical mixed effects models, we were able to account for groupings in our data, through the specification of random effects which recognizes that data coming from the same study and the same site will be more similar than ones from different studies and sites.

In order to address sampling bias, such as an overrepresentation of samples within certain biomes and realms, we modelled every realm and biome as a separate hierarchical linear mixed effects model. This avoids regularization of estimates of one biome, as influenced by another. By allowing estimates to vary independently for each realm, biome and response, we estimated independently for each without heavy sampling bias of one biome or realm influencing the estimate for an undersampled biome or realm. Realms and biomes that were better sampled have less uncertainty in estimates, than in those that were less sampled.

This accurately represents what is currently known of these systems, while highlighting important biome and realm-based gaps.

Despite these efforts, heterogeneity in samples, scales and regional coverage can certainly influence our results. We carefully checked the statistical modelling sections in the Methods to make sure that all relevant information was present and clear (Lines 592-658). Because this section is now right at the end of the manuscript, we made sure to mention inclusion of sampling effort variables in the brief description of the study given in the Introduction (Lines 135-146), while also in the Discussion describing potential issues with our data, how we tried to deal with them, and what the implications might be (Lines 426-448).

Recommendation
I recommend publication after minor revision, mainly to clarify potential biases, strengthen the discussion of uncertainty, and ensure figure and table legends are fully self-explanatory.

Thank you. We hope that the changes we have made in response to the comment above are satisfactory in these respects.

In addition, when revising the manuscript, we also made a number of changes to improve clarity and consistency. These include:

Major changes to Figures 2 & 3. By switching axes to have species richness and seed density on the x-axis, we could now put more modelled results on the same axis scale, as well as decompressing the axes somewhat. This allows a much easier comparison of the different aspects of seed bank diversity across ecosystems, while also making differences according to spatial scale and habitat degradation within ecosystems easier to see.

All Table and Figure captions have been checked and edited to improve clarity. Figures have now been formatted according to journal guidelines, ensuring that important labels are now at the maximum allowed font sizes.

We replaced the word ‘biome subtype’ with ‘ecoregion’ throughout the manuscript. While still a subcategory of biome according to our categorisation of ecosystems, we find this to be a more intuitive word, reflecting the fact that this part of the categorisation was based on the WWF ecoregion map. Otherwise, we have also worked hard to be consistent in our language, so that figure labels, captions, and related parts of the main text can be easily understood.

Reviewer #3 (Remarks to the Author):

I co-reviewed this manuscript with one of the reviewers who provided the listed reports. This is part of the Nature Communications initiative to facilitate training in peer review and to provide appropriate recognition for Early Career Researchers who co-review manuscripts.
Thank you. This sounds like a great initiative!
